# Temporal changes in emotional intelligence (EI) among medical undergraduates: a 5-year follow up study

**DOI:** 10.1186/s12909-020-02404-x

**Published:** 2020-12-09

**Authors:** Priyanga Ranasinghe, Vidarsha Senadeera, Nishadi Gamage, Miyuru Ferrari Weerarathna, Gominda Ponnamperuma

**Affiliations:** 1 Department of Pharmacology, Faculty of Medicine, University of Colombo, Colombo, Sri Lanka; 2 Department of Anatomy, Faculty of Medicine, University of Colombo, Colombo, Sri Lanka; 3 Department of Pathology, Faculty of Medicine, University of Colombo, Colombo, Sri Lanka; 4 Department of Medical Education, Faculty of Medicine, University of Colombo, Colombo, Sri Lanka

**Keywords:** Emotional intelligence, Medical undergraduates, Medical education, Time-related changes

## Abstract

**Background:**

Emotional intelligence (EI) is thought to play a significant role in professional and academic success. EI is important for medical personnel to cope with highly stressful circumstances during clinical and academic settings. The present prospective follow-up study intends to evaluate the changes in EI and their correlates among medical undergraduates over a five-year period.

**Methods:**

Data were collected in 2015 and 2020 at the Faculty of Medicine, University of Colombo, Sri Lanka. EI was assessed using the validated 33-item self-assessment tool, Schutte Self-Report Emotional Intelligence Test (SSEIT). In addition, socio-demographic details, students’ involvement in extracurricular-activities during undergraduate life, students’ satisfaction regarding the choice of studying medicine and plans to do postgraduate studies were also evaluated. A multiple-regression analysis was conducted among all students using percentage change in EI score as the continuous dependent variable, together with other independent variables (plan to do postgraduate studies, satisfaction in choice of medicine and extracurricular-activities).

**Results:**

Sample size was 170 (response rates–96.6%), with 41.2% males (*n* = 70). Mean EI scores at baseline among all students was 122.7 ± 11.6, and it had significantly increased at follow-up to 128.9 ± 11.2 (*p* <  0.001). This significant increase was independently observed in both males (122.1 ± 12.2 vs. 130.0 ± 12.4, *p* <  0.001) and females (123.1 ± 11.1 vs. 128.2 ± 10.3, *p* = 0.001). During follow-up, an increase in EI score was observed in students of all religions and ethnicities. Mean EI score also increased in all categories of monthly income, irrespective of the employment status or attainment of higher education of either parent. An increase in mean EI score during follow-up was observed in students irrespective of their engagement in or number of extracurricular-activities, they were involved. In the multiple regression analysis, being satisfied regarding their choice of the medical undergraduate programme (OR:11.75, *p* = 0.001) was the only significant factor associated with the percentage change in EI score.

**Conclusion:**

EI in this group significantly improved over 5-years of follow-up and was independent of gender, religion, ethnicity, socio-economic parameters and academic performance. Satisfaction in the chosen field was a significant predictor of the overall change in EI. Future studies are  needed to identify and measure factors responsible for improvement in EI among medical undergraduates.

## Background

Emotional intelligence (EI) is a domain of intelligence which has gained wide popularity over the last few decades. It could be simply defined as one’s ability to understand and regulate emotions of themselves and others [1]. Studies have shown that EI plays a significant role in professional and academic success than intelligence quotient (IQ) [2]. For example, academic performance based on grade point average (GPA) has a strong correlation with EI components [3]. Primary domains in EI include: self-management, self-awareness, relationship management and social awareness [2]. Since EI also encompasses traits, such as motivation and determination, it is logical to assume that people with a higher EI are likely to be more successful in life, with studies showing that it is a strong predictor of career success, independent of personality traits [4].

Medical students are in a unique position, as it is essential for them to be equipped with adequate interpersonal and intrapersonal skills to cope with highly stressful circumstances during clinical and academic settings [5]. Several independent studies from Belgium, Malaysia, Pakistan and Sri Lanka has shown that higher EI is associated with better academic performance among medical undergraduates (UGs) [6–9]. In addition, a higher EI was observed in those who had a higher level of self-satisfaction, whereas, self-perceived stress was lower in those with a higher EI among medical undergraduates [8]. Higher EI is also important for healthcare professionals, and is known to be associated with reduced stress [10], improved patient satisfaction [11], and higher levels of patient-centered care [12]. This makes EI training a crucial component in medical education [13], and different approaches have been used around the world for its incorporation into medical education curricula [14]. The present prospective follow-up study intends to evaluate the changes in EI with time (temporal change/improvement) and their correlates among medical undergraduates at Faculty of Medicine, University of Colombo over a five-year period.

## Methods

### Study population and sampling

This longitudinal prospective 5-year follow-up study was conducted at the Faculty of Medicine, University of Colombo, Sri Lanka. Faculty of Medicine, University of Colombo, is the 2nd oldest medical school in the South Asian region, with a mission to produce  healthcare professionals who are competent, compassionate and caring [15]. The five year curriculum includes different teaching methods and training strategies to achieve this objective, which would also contribute towards the enhancement of EI among its undergraduates [16]. Data were collected during two different study periods (October–November 2015 and July 2020). All students in the 2nd year of study who have completed studying the basic sciences (2nd year UGs, *n* = 205) were invited for the study (details of the above examinations are given below) in 2015. A further description about this cohort is published elsewhere [8]. All medical UGs who participated in the previous EI survey in 2015 (*n* = 176) were invited to participate in the follow-up study conducted in 2020. Informed written consent was obtained from the participants prior to data collection. Ethics approval for the study was obtained from the Ethics Review Committee, Faculty of Medicine, University of Colombo, Sri Lanka. The study is reported in accordance with the STROBE guidelines (Supplementary File 1).

### Study instrument, data collection and definitions

A self-administered questionnaire, consisting of two sections was used for data collection. The questionnaire was distributed, and data were collected online, identity was verified using student institutional emails, and duplicate entries were prevented. The questionnaire included two main sections. Section A included socio-demographic details of the respondents (age, area of residence, gender, religion, ethnicity, monthly family income, parents’ level of education, parents’ employment status and students’ involvement in extracurricular activities during undergraduate life). Furthermore, details about the participants’ satisfaction regarding the choice of studying medicine (satisfied/unsatisfied/not sure) and plans to do postgraduate studies were also collected. All parameters in section A were assessed at baseline, except participation in extracurricular activities, which was evaluated at follow-up. EI (Section B) of study participants was assessed using the validated 33-item self-assessment tool, Schutte Self-Report Emotional Intelligence Test [SEIT], which is the same questionnaire used in the previous survey [17]. Respondents rated themselves in each of the 33-items, according to a five-point Likert scale (1 = strongly disagree to 5 = strongly agree). Scores can range from 33 to 165 with higher scores characterizing higher EI. The percentage change of EI from baseline to follow-up was calculated by deducting follow-up EI score from baseline EI score.

Details of the medical curriculum at Faculty of Medicine, University of Colombo are presented elsewhere [16]. The medical undergraduate curriculum at the faculty spans 5 years, where the initial 1½ years of pre-clinical teaching is followed by 3½ years of clinical teaching. The entire curriculum is subdivided into 5 main streams; Introductory Basic Sciences Stream (IBSS), Applied Sciences Stream (ApSS), Clinical Sciences Stream (CSS), Community Stream (CS) and Behavioural Sciences Stream (BSS). The IBSS spanning the initial 1½ years focuses on teaching the basic sciences of Anatomy, Physiology and Biochemistry. The students are assessed at the end of the IBSS, with a barrier examination consisting of content from the three above mentioned subjects. Those who pass in the first attempt are graded as follows, based on the cumulative mark of the IBSS examination; ‘First Class’ ≥ 70 marks, ‘Second Class – Upper division’ 65–69 marks, ‘Second Class – Lower division’ 60–64 marks and ‘Pass’ 50–59 marks. A similar grading is given at the end of the ApSS and CSS. Hence, all the three streams described above (IBSS, ApSS and CSS), uses a cumulative mark to grade the students, with distinctions in respective subjects being offered to high performing students. The present study also evaluated the student’s performance at IBSS (initial main exam) and CSS (final main exam) examinations, including pass/repeat status, obtainment of classes and distinctions.

### Statistical analysis

Statistical Package for Social Sciences (SPSS) software, version 20.0 was used in the analysis of data. The variables were developed for analysis in SPSS and collected data were entered to a data sheet. Descriptive data are presented as percentages or as mean ± standard deviations. Significance of association was tested using Chi square test for categorical variables and Student’s dependent sample t-test for continuous variables. A multiple-regression analysis was also conducted among all students using percentage change in EI score as the continuous dependent variable, together with other independent variables, which were found to have significant association with EI in the univariate analysis, where a *p*-value of 0.10 was considered as the cut-off for variable inclusion. A similar regression was conducted independently in both males and females. In all analyses a p-value ≤0.05 was considered statistically significant.

## Results

### Socio-demographic characteristics

Sample size was 170/176 (response rates – 96.6%), with 41.2% males (*n* = 70). All of the responders were Sri Lankans, while all 6 non-respondents during follow-up at 5-years were identified as foreign students. The highest number of respondents (24.1%, *n* = 41) were residents of the Colombo district, the commercial capital of Sri Lanka. The ethnicity and religion of most students were Sinhalese (88.8%, *n* = 151) and Buddhism (82.4%, *n* = 140) respectively. When parental educational level was assessed, 44.0% (*n* = 68) of the fathers and 36.5% (*n* = 62) of the mothers had reached the level of higher education. Furthermore 89.2% (*n* = 141) of fathers and 50.0% of mothers (*n* = 85) were engaged in active employment at the time of the survey. Monthly family income was above 60,000 Sri Lankan Rupees (US$ 330) in the majority of respondents (54.7%, *n* = 93). Majority of the students indicated that they were involved in extracurricular activities during their stay at the university (71.2%, *n* = 121). These included sports (13.3%, *n* = 38), dancing, music and drama together (45.4%, *n* = 55), or participation in activities of different clubs/societies (78.3%, *n* = 94).

### Emotional intelligence: temporal changes and socio-demographic correlates

Mean EI score at baseline among all students was 122.7 ± 11.6, and it had significantly increased at follow-up to 128.9 ± 11.2 (*p* <  0.001). This significant increase was independently observed in both males (122.1 ± 12.2 vs. 130.0 ± 12.4, *p* <  0.001) and females (123.1 ± 11.1 vs. 128.2 ± 10.3, *p* = 0.001). There was no significant difference in the mean EI score between males and females, both at baseline (122.1 ± 12.2 vs. 123.1 ± 11.1, *p* = 0.580) and follow-up (130.0 ± 12.4 vs. 128.2 ± 10.3, *p* = 0.302). During follow-up, an increase in EI score was observed in students of all religions and ethnicities (Table 1). However, this increase was only significant in the Buddhist among religions (*p* <  0.001) and in Sinhalese among ethnicities (*p* <  0.001), the groups which comprised the largest sample sizes (Table 1). Mean EI score also increased in all categories of monthly income, although the increase was only significant in the LKR 20,000–40,000 and > LKR 60,000 categories (Table 1). Furthermore, a significant increase in mean EI score was noted in students, irrespective of the employment status or attainment of higher education of either parent (Table 1). This was independently observed in students of both genders. In addition, there was no significant difference between groups in the mean percentage change in EI from baseline to follow-up for gender, religion, ethnicity, monthly income, parental educational status and parental employment status (Table 1).

**Table 1:** Emotional Intelligence and Socio-Demographic Correlates

	Mean EI Score ± SD All students (*n* = 170)		*P* value*	Mean % change in EI score ± SD	Mean EI Score ± SD Males (*n* = 70)		Mean EI Score ± SD Females (*n* = 100)	
	Baseline	Follow-up			Baseline	Follow-up	Baseline	Follow-up
Gender								
Male (n = 70)	122.1 ± 12.2	130.0 ± 12.4	< 0.001	7.6 ± 15.3	NA	NA	NA	NA
Female (n = 100)	123.1 ± 11.1	128.2 ± 10.3	0.001	4.9 ± 12.6	NA	NA	NA	NA
Religion								
Buddhism (n = 140)	122.4 ± 11.2	128.9 ± 11.2	< 0.001	6.2 ± 13.3	122.0 ± 12.3	129.7 ± 12.2	128.3 ± 10.6	122.7 ± 10.5
Hinduism (*n* = 12)	122.3 ± 12.3	123.0 ± 10.4	0.905	10.4 ± 19.8	117.5 ± 15.8	122.8 ± 14.8	123.1 ± 8.7	124.8 ± 10.6
Christianity (n = 10)	119.7 ± 14.1	129.9 ± 10.8	0.160	1.8 ± 15.4	121.2 ± 9.8	128.5 ± 13.7	130.8 ± 9.6	118.7 ± 17.2
Islam (*n* = 6)	130.2 ± 10.6	138.5 ± 8.5	0.306	7.3 ± 14.2	122.3 ± 4.0	143.3 ± 7.2	133.7 ± 7.6	138.0 ± 8.9
Ethnicity								
Sinhalese (*n* = 151)	122.4 ± 11.5	129.0 ± 11.2	< 0.001	6.4 ± 13.8	122.3 ± 12.3	129.8 ± 12.1	128.5 ± 10.5	122.5 ± 11.0
Tamil (n = 12)	122.3 ± 12.3	123.0 ± 10.4	0.905	6.5 ± 13.1	117.5 ± 15.8	122.8 ± 14.8	123.1 ± 8.7	124.8 ± 10.6
Muslim (n = 7)	128.7 ± 10.4	136.1 ± 9.9	0.279	1.8 ± 15.4	122.3 ± 4.0	143.3 ± 7.2	130.8 ± 8.5	133.5 ± 11.6
Monthly Income								
< LKR 20,000 (n = 10)	125.9 ± 14.4	128.0 ± 13.9	0.771	3.2 ± 18.0	128.8 ± 14.2	125.6 ± 16.4	130.4 ± 12.2	123.6 ± 15.9
LKR 20,000–40,000 (*n* = 25)	117.9 ± 14.8	126.8 ± 13.5	0.029	9.2 ± 18.0	114.1 ± 18.0	130.3 ± 12.3	124.0 ± 14.3	120.9 ± 11.5
LKR 40,001–60,000 (*n* = 42)	123.6 ± 9.9	128.2 ± 9.8	0.064	4.4 ± 12.7	126.1 ± 9.1	128.6 ± 14.1	127.9 ± 6.2	122.1 ± 10.2
> LKR 60,000 (*n* = 93)	123.2 ± 10.8	129.9 ± 10.9	< 0.001	6.2 ± 12.6	121.9 ± 10.2	131.0 ± 11.5	129.1 ± 10.6	124.1 ± 11.2
Parents’ Employment Status								
Mother employed (n = 85)	122.4 ± 10.8	129.2 ± 11.0	< 0.001	6.4 ± 13.0	122.0 ± 10.4	130.8 ± 11.8	128.1 ± 10.5	122.6 ± 11.2
Mother unemployed (*n* = 85)	123.0 ± 12.3	128.6 ± 11.5	0.003	5.7 ± 14.7	122.1 ± 13.9	129.2 ± 13.1	128.2 ± 10.3	123.6 ± 11.2
Father employed (*n* = 141)	122.7 ± 11.1	128.8 ± 11.1	< 0.001	5.8 ± 13.7	122.0 ± 12.2	129.8 ± 11.9	128.0 ± 10.4	123.2 ± 10.3
Father unemployed (*n* = 29)	122.5 ± 13.7	129.5 ± 12.1	0.040	6.9 ± 14.8	122.2 ± 13.0	130.7 ± 15.8	128.9 ± 10.2	122.7 ± 14.4
Higher Education – Mother								
Yes (*n* = 62)	122.5 ± 10.9	129.1 ± 11.7	0.002	5.2 ± 12.4	123.2 ± 8.6	131.4 ± 13.3	127.9 ± 10.8	122.1 ± 12.0
No (*n* = 108)	122.8 ± 12.0	128.8 ± 11.0	< 0.001	6.6 ± 14.7	121.6 ± 13.5	129.4 ± 12.1	128.4 ± 10.1	124.0 ± 10.5
Higher Education – Father								
Yes (*n* = 68)	122.4 ± 10.7	129.6 ± 11.5	0.003	6.2 ± 13.3	120.8 ± 7.2	130.2 ± 12.8	126.7 ± 9.8	123.1 ± 12.0
No (*n* = 102)	122.8 ± 12.1	127.8 ± 10.8	< 0.001	6.0 ± 14.1	122.6 ± 13.8	129.8 ± 7.2	129.4 ± 10.7	123.0 ± 10.4

* - *p* Value for comparison between baseline and follow-up among all students; *EI* Emotional Intelligence, *NA* Not Applicable, *SD* Standard Deviation

### Emotional intelligence: academic performance and extracurricular activities

During follow-up an increase in mean EI score was observed in students who passed or repeated the IBSS and CSS examinations (Table 2). A similar increase was noted in students, irrespective of the grade they obtained in the IBSS or CSS examinations. However, this increase was only significant in those who obtained First Class or Pass grade at IBSS and in those who obtained Second Lower or Pass grade in CSS examination (Table 2). Furthermore, both students who did and did not obtain distinctions at these two examinations (IBSS and CSS), had an increase in the mean EI score from baseline to follow-up. An increase in mean EI score during follow-up was observed in students irrespective of their engagement in extracurricular activities, or the number of extracurricular activities they were involved (Table 2).

**Table 2:** Emotional Intelligence, Academic Performance and Extracurricular Activities

	Mean EI Score ± SD							Mean % change in EI score ± SD
	Baseline			Follow-up			*p* value^*^	
	All students (n = 170)	Males (n = 70)	Females (n = 100)	All students (*n* = 170)	Males (*n* = 70)	Females (*n* = 100)		
IBSS Examination								
Pass (*n* = 124)	123.1 ± 11.3	122.2 ± 11.8	123.8 ± 10.9	129.1 ± 11.6	130.7 ± 13.3	128.0 ± 10.1	< 0.001	6.0 ± 14.3
Repeat (*n* = 46)	121.4 ± 12.3	121.6 ± 13.6	121.3 ± 11.6	128.3 ± 10.4	127.8 ± 9.3	128.6 ± 11.1	0.003	6.2 ± 12.6
IBSS Examination Grade								
First Class (n = 19)	122.2 ± 10.1	120.0 ± 7.0	123.2 ± 11.3	134.3 ± 13.4	142.5 ± 13.0	130.5 ± 12.2	0.014	9.7 ± 15.1
Second Upper (*n* = 32)	124.0 ± 14.5	121.8 ± 15.6	125.4 ± 13.9	128.3 ± 9.1	128.4 ± 10.5	128.2 ± 8.3	0.264	4.4 ± 16.1
Second Lower (*n* = 30)	125.4 ± 9.0	125.1 ± 10.6	125.6 ± 8.3	129.6 ± 13.5	132.0 ± 16.7	128.3 ± 11.5	0.107	4.8 ± 14.1
Pass (*n* = 43)	121.3 ± 10.5	121.7 ± 11.4	121.0 ± 9.8	127.1 ± 10.5	128.2 ± 11.9	125.9 ± 9.0	0.004	6.6 ± 12.7
IBSS Examination Distinction								
Yes (n = 55)	124.1 ± 12.7	123.6 ± 14.2	124.4 ± 11.8	130.2 ± 11.6	131.0 ± 14.4	129.7 ± 9.7	0.015	6.2 ± 15.9
No (*n* = 73)	122.0 ± 10.0	121.5 ± 9.9	122.4 ± 10.1	128.1 ± 11.0	130.4 ± 12.5	126.3 ± 9.4	0.001	5.7 ± 12.9
CSS Examination								
Pass (n = 141)	123.0 ± 11.5	122.5 ± 12.2	123.3 ± 11.1	128.7 ± 11.0	129.8 ± 12.8	128.0 ± 9.7	< 0.001	5.7 ± 14.1
Repeat (*n* = 28)	121.0 ± 12.0	120.0 ± 13.1	121.9 ± 11.2	129.9 ± 12.5	130.8 ± 11.3	129.2 ± 13.8	0.003	8.1 ± 12.4
CSS Examination Grade								
First Class (n = 9)	122.3 ± 10.4	127.0 ± 5.6	121.0 ± 11.4	132.8 ± 12.6	140.0 ± 15.5	130.7 ± 12.2	0.089	9.4 ± 16.2
Second Upper (n = 43)	124.7 ± 12.0	119.1 ± 13.8	128.0 ± 9.5	128.2 ± 11.0	128.6 ± 14.0	128.0 ± 9.1	0.164	3.9 ± 14.5
Second Lower (*n* = 57)	121.7 ± 10.1	125.0 ± 9.5	119.9 ± 10.1	128.4 ± 11.1	130.3 ± 13.6	127.3 ± 9.6	0.003	6.3 ± 13.6
Pass (*n* = 31)	122.4 ± 13.2	122.2 ± 13.6	122.6 ± 13.2	128.6 ± 10.8	129.2 ± 11.1	127.7 ± 10.8	0.061	6.3 ± 14.5
CSS Examination Distinction								
Yes (n = 41)	123.8 ± 12.2	121.1 ± 13.9	125.5 ± 11.0	129.7 ± 11.6	131.3 ± 15.2	128.7 ± 8.6	0.038	6.0 ± 16.1
No (*n* = 99)	122.4 ± 11.1	123.0 ± 11.5	122.0 ± 10.8	128.2 ± 10.8	129.2 ± 11.9	127.5 ± 10.1	< 0.001	5.7 ± 13.3
Extracurricular activities								
Yes (*n* = 129)	123.0 ± 11.5	122.5 ± 12.4	123.5 ± 10.8	129.3 ± 11.5	130.2 ± 13.0	128.6 ± 10.1	< 0.001	7.1 ± 14.1
No (*n* = 41)	121.8 ± 11.7	120.6 ± 11.9	122.3 ± 11.8	127.9 ± 10.6	129.3 ± 10.4	127.3 ± 10.9	0.006	2.9 ± 12.5
Number of Extracurricular activities								
One (*n* = 56)	121.1 ± 11.9	122.8 ± 12.2	119.6 ± 11.6	130.2 ± 11.3	132.0 ± 12.5	128.6 ± 10.2	< 0.001	8.5 ± 13.4
Two (*n* = 51)	122.1 ± 13.3	120.8 ± 14.6	122.9 ± 12.6	128.1 ± 10.9	127.8 ± 13.6	128.3 ± 9.0	0.015	6.3 ± 15.6
Three (*n* = 22)	125.8 ± 10.6	121.8 ± 10.5	129.1 ± 9.9	131.4 ± 11.3	132.1 ± 12.6	130.9 ± 10.6	0.097	5.2 ± 12.4
Satisfaction with chosen field								
Satisfied (*n* = 111)	122.8 ± 11.8	122.0 ± 12.6	123.4 ± 11.3	130.6 ± 10.5	130.9 ± 11.8	130.5 ± 9.5	< 0.001	7.5 ± 14.4^#^
Not Satisfied (*n* = 19)	126.3 ± 11.9	127.9 ± 14.6	125.2 ± 10.1	120.0 ± 13.0	121.2 ± 15.7	119.1 ± 11.4	0.122	-4.2 ± 13.6^#†^
Not Sure (*n* = 40)	120.5 ± 10.2	119.4 ± 9.1	121.2 ± 11.1	128.3 ± 10.6	131.7 ± 11.2	126.0 ± 9.7	< 0.001	6.9 ± 9.8^†^
Plan to do postgraduate studies								
Yes (*n* = 119)	123.6 ± 11.4	123.3 ± 12.7	123.8 ± 10.4	128.7 ± 11.8	129.8 ± 12.7	127.9 ± 11.1	0.001	5.0 ± 13.8
No (n = 51)	120.5 ± 11.8	119.2 ± 10.6	121.5 ± 12.7	129.5 ± 9.9	130.4 ± 12.0	128.8 ± 8.3	< 0.001	8.4 ± 13.7

* - *p* Value for comparison between baseline and follow-up among all students; *CSS* Clinical Sciences Stream, *EI* Emotional Intelligence, *IBSS* Introductory Basic Science Stream, *SD* Standard Deviation; # *p* – 0.001; ^†^ *p* – 0.003

Ambition for postgraduate studies did not have an influence on the change in mean EI score from baseline to follow-up. However, with regards to their satisfaction in the chosen field, a significant increase in mean EI score was observed in those who were satisfied with their choice, while a non-significant decrease was observed in those who were not satisfied (Table 2). This is a finding which was also independently observed within gender. Furthermore, a significant percentage change in EI was also noted between these groups (7.5% vs. − 4.2%) (Table 2). However, there was no significant difference between groups in the mean percentage change in EI from baseline to follow-up for IBSS/CSS examination results, IBSS/CSS examination grade, obtaining IBSS/CSS distinctions or involvement in extracurricular activities.

### Results of the regression analysis

Table 3 summarizes the results of the binary logistic-regression analysis. The overall model demonstrated statistical significance (*p* = 0.006), the Cox & Snell R-Square and Nagelkerke R Square values were 0.095 and 0.126 respectively. The final results indicated that being satisfied regarding their choice of the medical undergraduate programme (OR: 11.75, *p* = 0.001) was the only significant factor associated with the percentage change in EI score. This was independently observed in both males and females (Table 3). However, ambition to do postgraduate studies and engagement in extracurricular activities were not associated with percentage change in EI score, in all students, males or females (Table 3).

**Table 3:** Results of the linear logistic regression analysis

	All students		Males		Females	
	Odds ratio (95% CI)	*p* Value	Odds ratio (95% CI)	*p* Value	Odds ratio (95% CI)	p Value
Plan to do postgraduate studies	−4.16 (−9.78–1.25)	0.131	−7.36 (− 17.48–2.77)	0.151	− 2.50 (− 8.92–3.92)	0.440
Satisfaction in choice of medicine	11.75 (4.70–18.79)	0.001	14.52 (1.63–27.41)	0.028	10.98 (2.47–19.49)	0.012
Extracurricular activities	0.20 (−5.55–5.95)	0.224	3.44 (−8.75–15.64)	0.573	−1.57 (−7.99–4.85)	0.627

*CI* Confidence Interval

## Discussion

To the best of our knowledge the present study is the first to explore temporal changes in EI, in a cohort of medical undergraduates. Our results show that EI in this group significantly improved over 5-years of follow-up, a finding which was independently observed in students of both genders. Furthermore, the improvement in EI was independent of religious backgrounds and ethnicities and although the magnitude of improvement varied between the different religions and ethnicities, these differences were not statistically significant. In addition, the improvement observed was independent of other measured socio-economic parameters such as, the family income, parental educational status and parental employment status. An improvement in EI scores were also observed, irrespective of their academic performance (pass/fail status, obtainment of classes and distinctions), participation in extracurricular activities and future ambitions to engage in postgraduate studies.

Furthermore, our results, including results from the multiple regression analysis show that satisfaction in the chosen field was a significant predictor of the overall change in EI in the present cohort. A finding which was independently observed in students from both genders. Previous cross-sectional studies have also demonstrated a close relationship between EI and satisfaction. For example, a study among nurses in Ghana demonstrated a significant positive relationship between EI and job satisfaction [18]. A similar relationship has been observed among newly qualified Malaysian dentists, where EI was the only statistically significant predictor of job satisfaction in multivariate analysis [19]. There are a number of reasons why high EI could lead to higher satisfaction. At the intrapersonal level, one would expect that individuals who understand their own moods and can use them effectively would have the skills and resources required to repair negative moods, regulate emotions, withstand workplace stress and increase satisfaction [20]. However, it is important to note that in previous cross-sectional studies higher EI was found to be a predictor of better satisfaction [8]. Conversely, our results demonstrate that satisfaction is an important predictor of temporal improvement in EI. Furthermore, in the present cohort, those who were not satisfied with the chosen field had higher EI at baseline in comparison to those who were satisfied, although the difference was not statistically significant. Hence, EI and temporal change in EI could have both a cause and effect relationship with satisfaction, where in addition to higher EI resulting in higher satisfaction, higher satisfaction could have an effect on temporal improvement in EI.

Studies from different countries and among students involved in different fields of study have shown that EI is closely linked with academic performance. For example, in a comparable cohort of Sri Lankan medical undergraduates, academic performance was better in those who were more emotionally intelligent, independent of gender [21]. Similar findings have been observed among students from Pakistan [22], India [23], Malaysia [6], Australia [24] and Iran [25]. However, it is important to note that these observations are derived from cross-sectional surveys. In the present study, the students were followed up over a period of 5 years and our results do not indicate any significant relationship between neither baseline nor follow-up EI score and academic performance. We did not observe any significant difference in baseline EI score and different levels of student achievements in the final year CSS exam (pass/repeat status, obtainment of classes and distinctions), a finding which was also independent of gender. Furthermore, as stated earlier irrespective of the level of academic performance EI score improved among students during the five-year follow-up period. However, before further conclusions are drawn, it is important to re-evaluate the association between EI and academic performance in longitudinal studies involving larger cohorts of students.

Unlike, IQ which is a relatively fixed construct of an individual, EI is a dynamic aspect of a person’s psyche and includes behavioral traits that can be improved with training. Previous studies have shown that training improves the EI of students [26], employees [27] and managers [28]. Our results also show that EI among medical undergraduates significantly improve with time, independent of gender and other measured socio-economic factors. The term EI encompasses several different but related facets such as; Adaptability, Assertiveness, Emotion perception (self and others), Emotion expression, Emotion management (others’), Emotion regulation, Impulsiveness (low), Relationships, Self-esteem, Self-motivation, Social awareness, Stress management, Empathy, Happiness, and Optimism [29]. Medical curricula, including that of Faculty of Medicine, University of Colombo, often include inputs from Behavioral Sciences and Medical Humanities, that also contain teaching/training activities that are aimed at improving the above facets of EI. Furthermore, clinical training involving encounters with patients in hospitals, would also contribute towards improving empathy and related aspects of EI among medical undergraduates. In addition, studies have also shown a positive relationship between a medical practitioner’s EI and satisfaction in the patients they treated [30]. A similar relationship has been observed among nursing staff working in surgical clinics [11]. The fact that EI can be improved independent of socio-demographic parameters and academic performance, as observed in the present study, will be helpful for medical educationalists in designing teaching/learning activities to further improve EI among medical undergraduates. This will not only help to improve their future career development, job satisfaction and well-being, but will also help to improve and strengthen, doctor-patient relationships and patient satisfaction.

The present study is the first prospective study to demonstrate an improvement in EI among medical undergraduates over a period of 5-years. The high response rate during follow-up (96.6%) was an additional strength of our study. However, sample size is a limitation and future large-scale longitudinal cohort studies are recommended to further validate our findings. Furthermore, since all medical students must undergo the same compulsory curriculum at the faculty, it was practically not possible to have a control group in the context of the present study. Thus we cannot directly say if the change noticed in EI across 5 years is the results of education and training or natural maturation. Future studies would need to take this into account, and have a comparable control group for more accurate comparisons. Furthermore, when same test is taken more than once performance can tend to improve, and could be one reason for the improvement noted. However, it is important to note that the 5 year gap between the two tests, would make the impact of this very minimal. For the same reason, the investigators did not conduct the EI tests on an annual basis. In addition, future studies also need to identify and measure factors responsible for improvement in EI among medical undergraduates that were not measured in the present study, such as inputs from medical humanities teaching and contribution from clinical training. This will help medical educationalists and curriculum designers to develop and strengthen such activities, to further improve EI among future medical practitioners.

## Conclusion

Emotional Intelligence in this group significantly improved over 5-years of follow-up, a finding which was independently observed in students of both genders and was independent of religion, ethnicity, socio-economic parameters, academic performance and participation in extracurricular activities. Satisfaction in the chosen field was a significant predictor of the overall change in EI. Future studies need to identify and measure factors responsible for improvement in EI among medical undergraduates in order to develop and strengthen activities, to further improve EI among future medical practitioners.

## Supplementary Information

**Additional file 1.**

## Data Availability

Data can be obtained via email by contacting the corresponding author.

## Abbreviations

EI: Emotional Intelligence
IQ: Intelligence Quotient
FOM, UOC: Faculty of Medicine, University of Colombo, Sri Lanka
GPA: Grade Point Average
LKR: Sri Lankan Rupees
US$: United States Dollars
